# Office Visitors

**Published:** 1907-04

**Authors:** 


### OFFICE VISITORS.

"Dees ist der docter, ees it?" Then he sat in a chair and moaned while he held his cheek between both hands. "Mine Gott in Himmel! Doc, der achin in my tooth ist seer badt alreddy, it swelledt mine face oudt und I yells met der pain. Vat you can do to heem?" Of course it was a case of abscess from the symptoms and he was put upon the proper treatment for relief and cure. When the tooth was in condition to fill we were halted in our operations by his desire to know what would be the charge for the completion of the case. We informed, so much would be for the treatment and so much would be for filling roots and crown. "Vell, look here, Doc, I ees no Rockerfeller, vat you tink ise 'got, tem row flats or tem sky scrapers? Nine. I vas von poor leetle baker, und I vas bake un tousan lofes of bread to pay dot. It vas too much Doc." After much argument he concluded to pay for the treatment, but would "poot oft der filling till I vas more besser oft." In a half hour we were summoned to the 'phone by a neighboring dentist who informed us that a "shopper" had come from us to him and that he had upheld our charge and gone him a dollar better. Another short space of time and a similar message came through the instrument from another practitioner. After several days when we had forgotten the incident our patient appeared again. "I say Doc, you vas a pooty goot fellow, vat you tink, I told you I vas coomin back ven I got besser oft. I buys von brandt new bread waggon, mit red paint, und gold letters und I sells more bread as never vas und I can pays dot leetle bill von der tooth. You fix heem goot, hein." When we had completed the case, the money paid and receipt given—which was carefully scrutinized, he informed us, "Yes Doc, you vas a pooty goot fellow. You vas besser as dose udder fellows, you pooty goot to der poor leetle baker. You was sheeper as der whole lot. I cooms to you next time again." When we inquired how he fitted his information, as to the other fellows' prices, with his declaration that he would return to us when he got better off, he replied. "Vell ven I vent to der odders to get der prices und get posted, youst to get posted, Doc, I drofe oop in mine new wagon, und dem fellows say rite away, dot vas one reech baker, I vas go for heem, und der prices vas, vun, two, tree tollers more as you. Yaas, you vas pooty goot, I tanks you. But ven I gets der toochache vudst more, I go by mine foots alreddy und no more een der brand new vaggin.

